# Clinical validation of a commercially available deep learning software for synthetic CT generation for brain

**DOI:** 10.1186/s13014-021-01794-6

**Published:** 2021-04-07

**Authors:** Minna Lerner, Joakim Medin, Christian Jamtheim Gustafsson, Sara Alkner, Carl Siversson, Lars E. Olsson

**Affiliations:** 1 Radiation Physics, Department of Hematology, Oncology, and Radiation Physics, Skåne University Hospital, Lund, Sweden; 2 Department of Translational Medicine, Medical Radiation Physics, Lund University, Malmö, Sweden; 3 Department of Clinical Sciences Lund, Oncology and Pathology, Lund University, Lund, Sweden; 4 Clinic of Oncology, Department of Hematology, Oncology and Radiation Physics, Skåne University Hospital, Lund, Sweden; 5 Spectronic Medical AB, Helsingborg, Sweden

**Keywords:** MRI-only, Brain, Synthetic CT, Radiotherapy, Glioma, Brain metastases

## Abstract

**Background:**

Most studies on synthetic computed tomography (sCT) generation for brain rely on in-house developed methods. They often focus on performance rather than clinical feasibility. Therefore, the aim of this work was to validate sCT images generated using a commercially available software, based on a convolutional neural network (CNN) algorithm, to enable MRI-only treatment planning for the brain in a clinical setting.

**Methods:**

This prospective study included 20 patients with brain malignancies of which 14 had areas of resected skull bone due to surgery. A Dixon magnetic resonance (MR) acquisition sequence for sCT generation was added to the clinical brain MR-protocol. The corresponding sCT images were provided by the software MRI Planner (Spectronic Medical AB, Sweden). sCT images were rigidly registered and resampled to CT for each patient. Treatment plans were optimized on CT and recalculated on sCT images for evaluation of dosimetric and geometric endpoints. Further analysis was also performed for the post-surgical cases. Clinical robustness in patient setup verification was assessed by rigidly registering cone beam CT (CBCT) to sCT and CT images, respectively.

**Results:**

All sCT images were successfully generated. Areas of bone resection due to surgery were accurately depicted. Mean absolute error of the sCT images within the body contour for all patients was 62.2 ± 4.1 HU. Average absorbed dose differences were below 0.2% for parameters evaluated for both targets and organs at risk. Mean pass rate of global gamma (1%/1 mm) for all patients was 100.0 ± 0.0% within PTV and 99.1 ± 0.6% for the full dose distribution. No clinically relevant deviations were found in the CBCT-sCT vs CBCT-CT image registrations. In addition, mean values of voxel-wise patient specific geometric distortion in the Dixon images for sCT generation were below 0.1 mm for soft tissue, and below 0.2 mm for air and bone.

**Conclusions:**

This work successfully validated a commercially available CNN-based software for sCT generation. Results were comparable for sCT and CT images in both dosimetric and geometric evaluation, for both patients with and without anatomical anomalies. Thus, MRI Planner is feasible to use for radiotherapy treatment planning of brain tumours.

**Supplementary Information:**

The online version contains supplementary material available at 10.1186/s13014-021-01794-6.

## Background

Due to its excellent soft tissue contrast, the use of Magnetic Resonance Imaging (MRI) is considered standard for radiotherapy planning in treatment of brain tumours [1]. However, CT images are still used in the workflow since they provide information about electron densities in various tissues, expressed in Hounsfield units (HU), which are needed for dose calculations performed by the treatment planning system. As a result, the MR images need to be registered to the CT images. This process introduces an undesired systematic geometrical uncertainty in the treatment planning process, which for brain images has been found to be in the order of 2 mm [2]. In order to avoid this registration uncertainty, the use of a single imaging modality would be advantageous. This was recently pointed out for stereotactic brain radiotherapy [3]. If information regarding various electron densities in the patient could be provided from MR images, the CT examination could be excluded from the workflow, thereby avoiding the image registration uncertainty and enabling an MRI-only workflow.

The development of methods for generating images with electron density information (HU) from MR images of different parts of the body has gained considerable attention, and the subject has recently been the topic of two major review papers [4, 5]. The information from MR images acquired with one or several MRI pulse sequences are converted into images representing HU, often referred to as pseudo CT or synthetic CT (sCT) images. The latter nomenclature will be used in this paper. Both review papers [4, 5] grouped the sCT generation methods into three main categories: bulk density assignment, atlas-based techniques, and voxel-based techniques. All three methods have been applied to the brain region with acceptable results but they are marred by certain limitations [5]. For the bulk density method, one limitation is the necessary segmentation of bone prior to sCT generation [6], which often requires manual input. The atlas-based methods are limited by the capability to handle patients with anatomical anomalies [7–9]. Atypical anatomy may not be a problem for voxel-based methods. However, the voxel-based methods frequently rely on the use of MR images from multiple sequence acquisitions [10], which results in extended scanning time and increased risk for patient motion. In addition, time-consuming manual segmentation of bone or air is often needed also for this method.

Recently, an additional category of methods for sCT generation for the skull was suggested [11]. The new method is based on deep learning using convolutional neural networks (CNN). Deep learning is a sub field of machine learning, where algorithms are developed to solve problems by learning from data and experience. The goal is to create models that can be trained to predict and produce new and accurate information from previously unseen data and the proposed technique has numerous of applications in medical imaging [12]. Deep learning differs from the other sCT methods since it relies on a dedicated elaborate machine learning training procedure in which matching or independent data sets of CT and MR images are fed into the model construction.

There are several examples of recent studies using deep CNN for generating sCT images of the skull. Various computing techniques have been applied and the sCT images have been evaluated against CT images with respect to HU and dosimetric accuracy for treatments of brain tumours or metastases. Reported results show differences in mean absolute error of HU between 55 and 85 HU [11, 13–17] and dosimetric differences between sCT and CT below 1% in the planning target volume [14, 15]. Quantitative evaluation of the overlap in bone for sCT and CT images using dice similarity coefficient (DSC) resulted in a DSC of 0.85–0.98 [13–15].

One aspect that has received less attention in previous studies using deep learning methods is anatomical anomalies such as bone resection due to surgery. Since deep learning methods allow for extensive data augmentation and typically generalizes much better than other methods, the network may be robust also to features occurring rarely in clinical data. Patients with primary brain tumours or brain metastasis often have received surgery prior to the start of radiotherapy [18, 19], and as a result the anatomy of the skull may diverge from the normal shape. A correct depiction of the skull is important for accurate treatment planning and delivery. In particular, the small anomalies, e.g. missing skull bone, are attractive anatomical landmarks when visually verifying the automatic registration during patient positioning. If the sCT-generation software cannot handle post-operative cases, patients may have to be excluded from the use of this technique. Hence, an accurate result of the anatomical anomalies from the sCT-generation method is important for a more general clinical implementation of an MRI-only radiotherapy planning workflow for brain.

The aim of this study was to evaluate the recently released software MRI Planner (Spectronic Medical AB, Helsingborg, Sweden), which utilizes the deep learning based Transfer Function Estimation (TFE) algorithm to generate sCT from MR-images of the skull, head-neck and pelvic regions [20]. The dosimetric and geometric accuracy for sCT images compared to conventional CT images was studied for patients with brain tumours, including anatomical anomalies due to pre-irradiation surgery. Finally, patient positioning using sCT images as references was investigated.

## Method

### Study design

This study was a prospective, non-invasive study approved by the regional ethics review board (dnr: 2018/445). Inclusion criteria were patients with glioma or brain metastases referred to MR and CT treatment planning examinations prior to external radiotherapy of the brain. The patients received oral and written information prior to study inclusion and signed a written consent if participating. Study participation did not affect treatment prescription and routine clinical workflow was followed.

### Patient data and imaging

The population included 20 consecutively recruited patients with prescribed treatment for glioma (n = 10) or one or several brain metastases (n = 10). 14 of the patients had parts of the skull bone missing due to surgical procedures prior to radiotherapy. The largest piece of resected bone in this set of patients was 3 cm, but the majority of the patients had drill holes of approximately 1 cm in diameter (for details please see Table 2). The mean age of the patients was 68 ± 9 years (range: 42–81 years).

Individual neck support and three point hybrid head immobilisation masks (Orfit Industries NV, Wijnegem, Belgium) fixated the patient during CT and MR examinations, and in the following radiotherapy treatment sessions. CT images were acquired using a Siemens Somatom Definition AS + (Siemens, Erlangen, Germany) with tube voltage 120 kV and 2 mm slice thickness with pixel sizes varying from 0.7 × 0.7 mm^2^ to 1.0 × 1.0 mm^2^. MR images were acquired within a few hours after the CT examination using a 3 T GE Discovery 750 W (Software version DV26.0-R03-1831.b, GE Healthcare, Chicago, Illinois, USA). During MR examination, a flat table top and 6 channel receiver flex coils were used.

Dixon MR images (in-phase, out-of-phase, water and fat) were required by the sCT generation software. Hence, a 3D IDEAL Dixon fast spoiled gradient echo (SPGR) acquisition sequence (Table 1), was added prior to contrast agent injection in the clinical MR protocol, extending the original 20 min protocol with 4.5 min. Slice thickness was 2 mm and in-plane resolution was 1.1 × 1.1 mm^2^. The Dixon output images were automatically reconstructed in-phase, out-of-phase, water and fat images. The Dixon sequence was modified to also produce a patient specific B0 distortion map, enabled by using a GE Healthcare customer variable (CV) parameter option in the sequence. Patient specific distortion was reduced by choosing a bandwidth of 744 Hz/pixel (440 Hz being the difference in resonance frequency between fat and water at 3 T) [21].

**Table 1:** Scanning parameters of the MRI-only specific sequences

Scanning parameter	3D IDEAL Dixon Fast SPGR	ZTE
Scanning time	4 min 30 s	21 s
Number of slices	116	20
Slice thickness	2 mm	4 mm
Slice gap	0 mm	0 mm
Bandwidth	744 Hz/px	434 Hz/px
Echo times	2.18, 2.97 & 3.76 ms	0 ms
Number of echo times	3	1
3D distortion correction	Enabled	Enabled
Field of view	240 mm × 240 mm	280 mm × 280 mm
Scanned voxel size	1.1 mm × 1.1 mm	1.5 mm × 1.5 mm
Scan matrix	224 × 224	192 × 192
Reconstructed voxel size	0.5 mm × 0.5 mm	1.1 mm × 1.1 mm
Reconstructed matrix size	512 × 512	256 × 256

The vertical position of the treatment couch relative to the isocenter is a required parameter in the treatment planning system (TPS) to allow for accurate absorbed dose calculations due to attenuation in the couch. In the conventional workflow the couch is visible in CT images and easily handled in the TPS. However, for the MR Dixon images the field of view did not cover the couch. Furthermore, due to the used echo times in the Dixon sequence the couch itself did not yield any substantial MR signal. Instead, in order to identify the vertical position of the treatment couch a Zero Echo Time (ZTE) sequence with ultrashort echo time (uTE) and a total scan time of 21 s was added. The position of the identified treatment couch in the ZTE images was validated against its position in the CT images, as part of preparing for a clinical MRI-only treatment workflow.

### sCT generation

To generate sCT images, a pre-release of the CE-marked software MRI Planner (v 2.2, Spectronic Medical AB, Helsingborg, Sweden) was incorporated to the clinical workflow through a cloud based service. Input files were the four MR-Dixon images (in-phase, out-of-phase, fat and water), which were exported directly from the MR-platform. The MRI Planner uses a high-resolution residual three dimensional deep CNN, with the TFE-algorithm, as described by the vendor [20]. Part of the training data set was obtained within a pre-study of this project, including several post-surgical cases with bone resection. There was no overlap between the training data and the data evaluated in this study. The MRI Planner software was recently evaluated for head and neck patients [22], where further details about the sCT generation method can be found.

The exported DICOM files were automatically pseudo-anonymized before leaving the hospital network and labelled with an individual key identifier. The sCT generation time was 4–6 min [20]. Upon return of the generated sCT the cloud link software restored the patient information to the images. The sCT images were rigidly registered to the CT images using bone match in six degrees of freedom (X, Y, Z, pitch, roll and rotation) with a registration box including the whole head and a caudal cut-off below the base of the brain. Finally, the sCT images were resampled to the resolution of the CT before import to the treatment planning system.

### Geometric distortion

Phantom measurements were carried out prior to patient inclusion to determine the impact of system dependent geometric distortion, due to main magnetic field inhomogeneities and gradient non-linearities. For this purpose, the GRADE phantom for geometric distortion measurements (Spectronic Medical AB, Helsingborg, Sweden) [23, 24] was imaged using the Dixon sequence with the same acquisition parameters as in Table 1, except for the field of view which was set to 500 × 500 mm^2^ and the scan matrix of 512 × 512 to fit the phantom size. The phantom contains approximately 1200 spherical markers which were automatically evaluated relative to a control template.

Patient specific distortion, due to chemical shift and susceptibility effects, are manifested as geometric distortions in the frequency encoding direction [25], defined in the anterior/posterior direction for the used Dixon sequence. To estimate the patient specific distortion, each patient’s B0 distortion map was evaluated voxel-wise. Each pixel in the distortion map contained the deviation in resonance frequency compared to the centre frequency in Hz, and was recalculated to mm absolute distortion (d), according to the following equation:

1 $$d = \frac{{\left| {\Delta B_{0} } \right|}}{BW} \cdot pixel\,size$$

The bandwidth (BW) was expressed in Hz/pixel and the pixel size corresponded to one side of the isotropic pixel in mm. The geometric distortion was calculated for three regions; brain, bone and air within the body contour of the patient. The teeth and jaw areas were excluded as these regions did not intersect with the radiation beam path. Bone segmentation was extracted from CT images for voxels of 250 HU and above while air was segmented for voxels less than − 900 HU. The binary masks were overlaid on the distortion maps.

### Treatment planning

For each patient a volumetric modulation arc therapy (VMAT) treatment plan was clinically optimised on the CT images in the Eclipse treatment planning system (TPS) (v. 13.6.23, Varian Medical Systems, Palo Alto, CA, USA). The VMAT plans contained 1–4 arcs to be delivered on Varian TrueBeam. For more details, see Table 2. Relevant organs at risk (OAR) in relation to target position were defined and all radiotherapy structures delineated on the CT images, except for the body contour, were then transferred to the sCT images. A new body structure for the sCT was automatically generated at image import. Absorbed dose was calculated using the anisotropical analytical algorithm (AAA, 13.6.23) with 1.0 × 1.0 mm^2^ or 2.5 × 2.5 mm^2^ grid size. Only HU values of voxels within the body contour and the assigned HU values within the couch structure were included in the dose calculation. The original treatment plan was recalculated on the sCT images, using identical plan parameters keeping the number of monitor units fixed.

**Table 2:** Patient overview with details about treatment, tumour location and bone resection

Patient	Glioma (G)/metastasis (M)	Prescribed dose [Gy]	Beam configuration	PTVvol [cc]	Bone resected (yes/no)	Max d* [mm]	Tumour location
1	M	25.0	2 arcs	31	N	–	Right thalamus
2	G	34.0	2 arcs, NC	408	Y	10	Right frontal/temporal
3	M	30.0	1 arc	5	Y	10	Left frontal
4	M	30.0	3 arcs, NC	10	Y**	10	Right occipital
5	G	40.05	1 arc	181	Y	10	Left frontal/parietal
6	G	40.05	2 arcs	448	Y	10	Right temporal
7	G	40.05	2 half arcs	186	Y	10	Left occipital/temporal
8***	M	30.0	2 arcs	21	Y	20	Cerebellum
9	M	30.0	1 arc	41	N	–	Right frontal
10***	M	30.0	2 arcs	2	N	–	Left occipital
11	G	60.0	4 arcs, NC	439	Y	10	Bilateral frontal/temporal
12	G	34.0	2 half arcs	314	Y	10	Left frontal/temporal
13	M	24.0	2 arcs	63	N	–	Left temporal
14	G	60.0	2 arcs	179	Y	10	Left frontal/temporal
15	G	60.0	2 half arcs	168	Y	10	Left temporal/occipital
16***	G	60.0	2 arcs, NC	430	Y	30	Left hemisphere
17	M	30.0	2 arcs	16	N	–	Left parietal
18	G	25.0	2 arcs	318	Y	10	Left hemisphere
19	M	30.0	2 arcs, NC	21	Y**	10	Right occipital
20	M	30.0	3 arcs, NC	25	N	-	Right parietal

Patients identified as having targets in complex regions were number 2, 6, 8, 11, 14, 16 and 18

^*^Max d refers to the maximum diameter of the bone resection. One patient may have more than one resection area, in which case the largest one is presented

^**^Bone resection from previous surgery, not adjacent to the PTV evaluated in this study

**Fig. 1:**
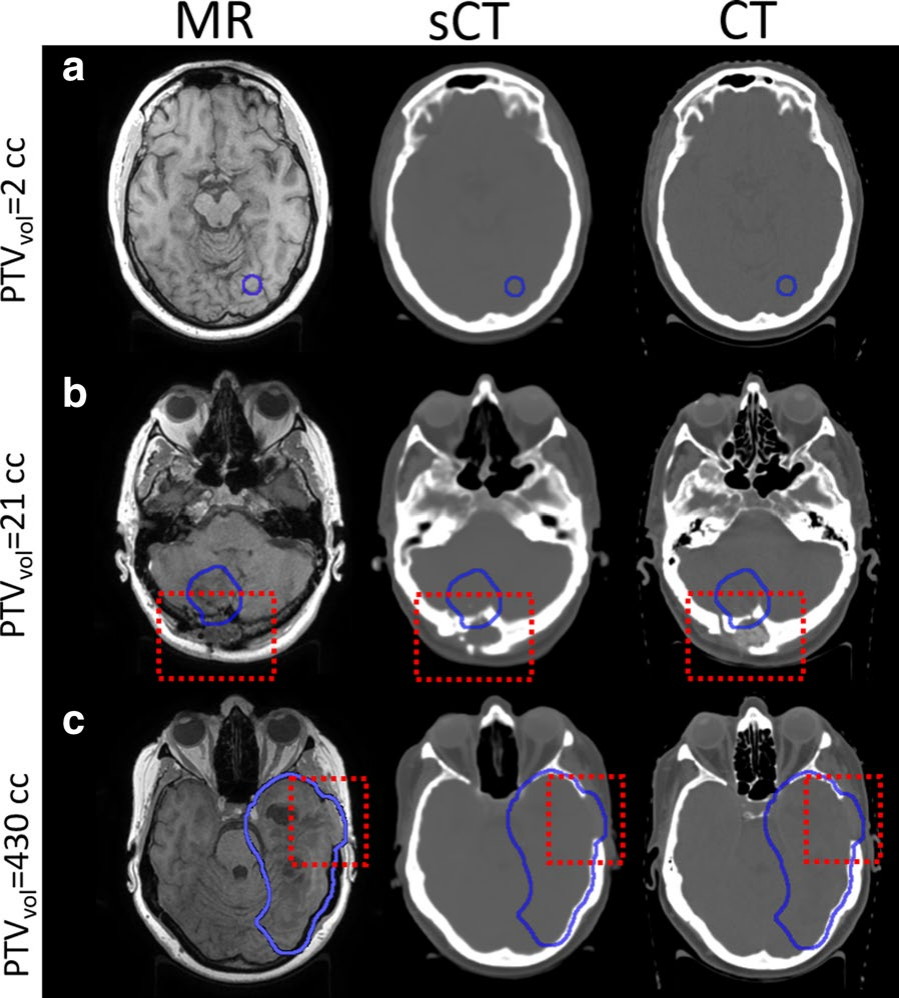
Transversal MR-Dixon (in-phase), synthetic CT and CT images for three patients (**a**–**c**) with PTV structures displayed in blue and regions of bone resection due to surgery within the red box (patient **b**, **c**). The volumes of the PTVs are displayed in the leftmost column

^***^Three of the patients are presented in Fig. 1 to represent the range of tumour locations and sizes; patient 10 corresponds to patient A, patient 8 corresponds to patient B and patient 16 corresponds to patient C

### sCT evaluation

The HU of the sCT images were evaluated based on pixel-to-pixel comparison to the CT images using mean absolute error (MAE) and mean error (ME) in three regions; within the intersection of the body contours (denoted body), brain and bone (excluding teeth and jaw). Dice similarity coefficient (DSC) was used to evaluate overlap in bone in the sCT and CT images with bone segmentation done at threshold 250 HU. All evaluations were carried out in MICE Toolkit (v. 2020.2.1 (Beta), Nonpi Medical, Umeå, Sweden), R (v. 4.0.0, R Foundation for Statistical Computing, Vienna, Austria) [26] or in Eclipse TPS.

### Dosimetric evaluation

The prescribed dose ranged between 24 and 60 Gy (2–10 Gy/fraction) over the patient population. More details are found in Table 2. Due to the large variations in fraction dose, all dosimetric results are presented as percentage of the prescribed dose.

Treatment plans for all patients were initially evaluated on the same criteria as in the clinical workflow. The analysis included dose volume histogram parameters for the planning target volume (PTV) and organs at risk (OAR), which were patient individual due to different target positions. Dose differences were evaluated using a Wilcox rank sum test. Separate statistical evaluation of the dose differences was performed for a subgroup of seven patients who were identified as having targets in or near complex regions, such as the nasal cavity (Table 2, patients # 2, 6, 8, 11, 14, 16 and 18). 3D global gamma analysis was performed of the full dose grid (cut-off dose at 15%) at 3%, 3 mm, 2%, 2 mm and 1%, 1 mm, comparing the dose distributions on sCT and CT images for all patients.

### Anatomical anomalies due to surgery

Patients who had undergone surgery prior to radiotherapy were further evaluated with focus on the skull bone resection. Volumes of interest (VOI) were manually delineated in the slices covering the resected bone region, with an added margin of 1 cm. MAE for bone and DSC were then calculated within the individual VOI for each of these patients.

### Patient set-up verification

To evaluate the feasibility of a complete MRI-only workflow, the cone beam CT (CBCT) images from one fraction of the conventional treatment for each patient were retrospectively registered to the sCT and CT images, respectively. Registration was performed in the TPS, using rigid bone registration (200–1700 HU) in six degrees of freedom. The registration box included the whole head, and a caudal cut-off at the skull base. Since the CT and sCT images were already in the same frame of reference, the results from the image registration of CT-CBCT could be subtracted from sCT-CBCT and compared separately for each degree of freedom.

## Results

### Geometric evaluation of MRI for sCT generation

The mean geometric distortion was 0.3 mm within a radius of 15 cm from the MRI isocenter obtained for the system related distortion, as measured using the GRADE phantom. The corresponding maximum distortion within 15 cm was 1.1 mm.

The average patient specific geometric distortion in the frequency encoding direction for the whole brain, air and bone was estimated to 0.07 ± 0.04 mm, 0.16 ± 0.06 mm and 0.10 ± 0.03 mm, respectively. The corresponding maximum distortions within the patient population were 0.9 mm, 1.3 mm and 1.2 mm, for brain, air and bone respectively. Finally, the maximum distortion within the 99% percentile was 0.9 mm or less for all patients and regions.

### ZTE evaluation

A representative case of a ZTE image is presented in the Additional file 1: Fig. E1. The vertical position of the treatment couch was successfully identified in the ZTE images within 0.1 ± 0.2 cm compared to its position in the CT images for the evaluated patients.

### sCT evaluation

Patients included in the study had PTV sizes in the range 2–448 cm^3^. Representative cases of MR-sCT-CT images for three patients (A-C) with different PTV sizes and tumour locations are shown in Fig. 1. Patient B and C had regions of skull bone resection due to surgery. The corresponding representative images for all patients can be found in the supplementary material (Additional file 1: Figs. E2–E5).

The average MAE and ME for the studied patient population are presented in Table 3. The ME shows that HU-values in the sCT images within the patient population were underestimated for bone, whereas the HU agreed well for the soft tissue in the brain. Average DSC was 0.92 ± 0.01 [0.90–0.94].

**Table 3:** Overall statistics of mean absolute error (MAE) and mean error (ME) comparing sCT and CT images for body, brain and bone. The results for bone are also presented separately for bone resected patients (n = 14) and non-resected patients (n = 6)

	Body	Brain	Bone	Bone (Resected)	Bone (Non-resected)
Number of patients	20	20	20	14	6
MAE [HU]					
Mean ± 1 S.D	62.2 ± 4.1	9.5 ± 0.7	173.8 ± 18.2	176.5 ± 18.8	164.1 ± 14.4
Range	56.2–70.4	8.3–11.2	144.3–208.0	153.1–208.0	144.3–179.7
ME [HU]					
Mean ± 1 S.D	− 5.6 ± 4.6	1.3 ± 2.0	− 41.9 ± 18.3	− 40.0 ± 18.3	− 46.3 ± 19.4
Range	− 13.5–1.5	− 2.7–4.5	− 74.8-(− 16.7)	− 74.8-(− 16.7)	− 70.0-(− 22.2)

### Dosimetric evaluation

**Fig. 2:**
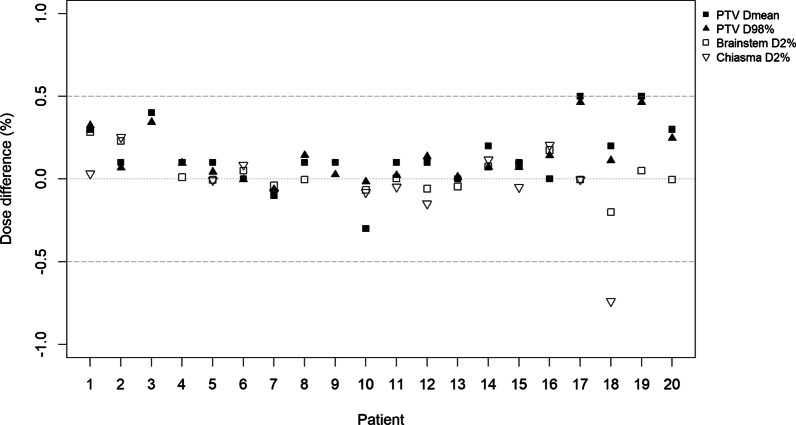
Deviations in percentage between dose distributions calculated on sCT and CT images (sCT-CT) for PTV, brainstem and chiasma

For the evaluated DVH parameters, the absorbed dose deviations between sCT and CT calculated treatment plans were within 0.5% except for one value at 0.7% (Fig. 2). The OAR doses are included for those patients where the structure was delineated (chiasma: n = 13, brainstem: n = 18). The average deviation for Dmean (PTV) and D98% (PTV) ± 1 S.D. [range] was 0.1 ± 0.2% [− 0.3 to 0.5%] and 0.1 ± 0.2% [− 0.1 to 0.5%], respectively. The corresponding results for D2%(brainstem) was 0.0 ± 0.1% [− 0.2 to 0.3%], and for D2%(chiasma) 0.0 ± 0.2% [− 0.7 to 0.3%]. None of these are statistically significant (*p* > 0.05). Neither for the sub group of patients with targets near complex regions compared to the patients with targets in non-complex regions were there any statistically significant difference (*p* > 0.05) in absorbed dose.

The 3D global gamma evaluation of the full dose distribution with 15% dose cut-off at three different dose and distance criteria was performed for all patients (Table 4). Pass rate for gamma evaluation (1%, 1 mm) of the voxels within the PTV was 100.0 ± 0.0% [99.9–100.0%].

**Table 4:** Global gamma pass rates comparing the sCT and CT dose distributions using a 15% dose cut-off

Gamma criteria	Gamma pass rate ± 1 S.D. (%)	Range (%)
3%, 3 mm	100.0 ± 0.0	99.9–100.0
2%, 2 mm	99.8 ± 0.2	99.1–100.0
1%, 1 mm	99.1 ± 0.6	97.9–99.8
1%, 1 mm (PTV)	100.0 ± 0.0	99.9–100.0

### Anatomical anomalies due to surgery

A subgroup of 14 patients had regions of bone resection due to surgery prior to radiotherapy. The sub-analysis resulted in average MAE of 176.5 HU ± 18.8 HU [153.1–208.0 HU] for bone in the whole skull and 222.0 HU ± 59.8 HU [99.9–359.2 HU] for bone in the volume surrounding the resection area (VOI). The DSC calculated for the whole skull averaged over the 14 patients was 0.92 ± 0.01 [0.90–0.94] compared to 0.90 ± 0.04 [0.81–0.95] in the VOI. For comparison, the remaining non bone resected six patients had an average MAE of 164.1 HU ± 14.4 HU [144.3–179.7 HU] for bone and average DSC of 0.93 ± 0.01 [0.90–0.94].

### Patient set-up verification

**Fig. 3:**
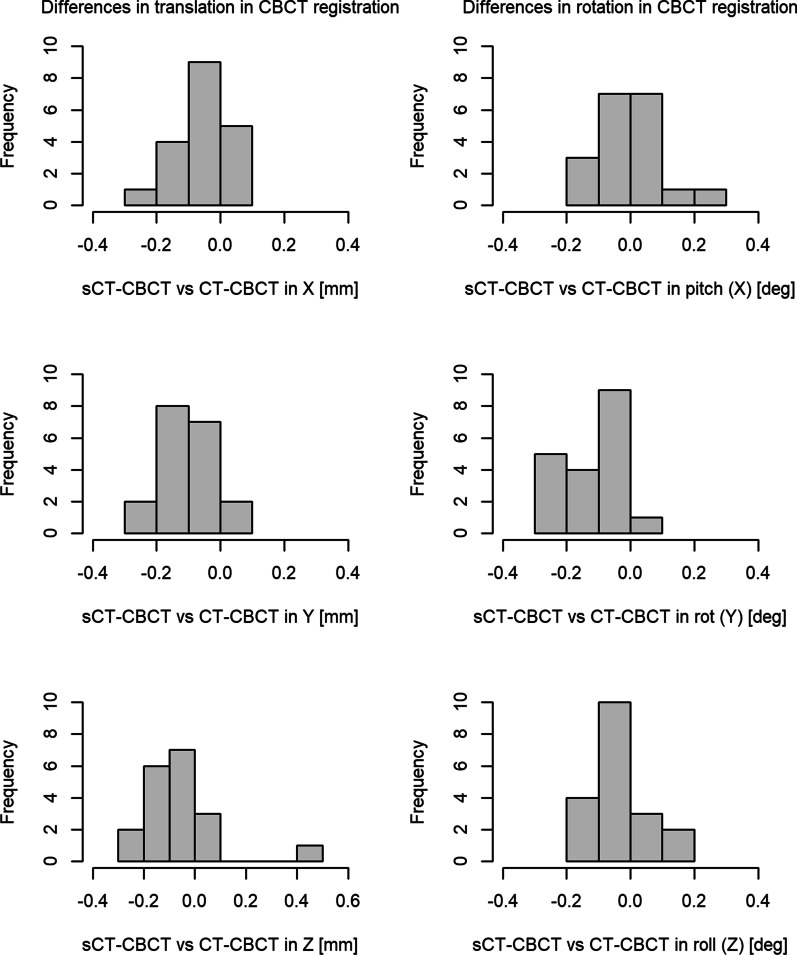
Difference in image registration results between sCT-CBCT and CT-CBCT in translations (left column) and rotations (right column). The X, Y and Z axis correspond to the following translations: X = left to right, Y = anterior to posterior and Z = superior to inferior. The histogram cells include their right-hand endpoint. Bin sizes are 0.1 units

CBCT images were available for 19 out of the 20 patients. CBCT images were unavailable for one patient as treatment was re-planned to a different machine with a different imaging modality (TomoTherapy). The differences between the image registrations sCT-CBCT and CT-CBCT for each degree of freedom are presented in Fig. 3.

## Discussion

In this study, a commercially available software for sCT generation was evaluated. The study results confirm that MRI Planner can generate adequate sCT images for radiotherapy treatment planning of brain tumours. The software could also provide sCT images for those patients in this study who had bone resection volumes due to surgery prior to radiotherapy. This is to the best of our knowledge the first evaluation of a commercially available sCT generation method that successfully handles patients with bone resection in the skull.

To verify that requirements on geometric integrity of MR images in a radiotherapy workflow were fulfilled [21, 25], geometric distortion in the Dixon images, from which the sCT images were generated, was investigated. Within a radius of 15 cm from the MR isocenter, a radius that covered the size of the skull for all patients, phantom measurements showed 0.3 mm mean geometric distortion and a maximum value of 1.1 mm. The patient specific evaluation, performed for each patient, yielded a mean absolute distortion for all investigated regions below 0.2 mm. The largest patient specific distortion was correlated to air cavities, as expected [21], where a maximum of 1.3 mm was found. The method to assess patient specific distortion reported in this study also included effects from B0 deviations in the MRI system itself, resulting in an overestimation of the patient specific distortion. Nevertheless, the maximum distortion found was small and we could conclude that there was no need to separate these effects from each other in this study. The effects of the system and patient specific geometric distortions present in this study were of no clinical concern [27], as also demonstrated in a previous head-and-neck study [28]. The SRS cases are more sensitive to distortions due to small or no treatment margins, but deviations less than one pixel (1 mm) for MR images used for sCT generation, as shown here, are yet of minor clinical concern. Finally, due to the fact that geometric distortions are scanner specific, each clinic needs to evaluate this prior to implementing MRI-only workflows.

Dosimetric evaluation of the treatment plans recalculated on sCT and compared to the original CT-based absorbed dose distribution, resulted in average absorbed dose difference in PTV mean dose of 0.1 ± 0.2%, with maximum deviations of 0.5%. The worst overall observed dose deviation was − 0.7% in D2% in chiasma for one patient, however the corresponding mean dose difference was only − 0.2%. Seven of the patients were identified to have targets in complex regions, for example close to the nasal cavity and sinuses. However, statistical testing showed no significant difference (*p* > 0.05) in absorbed dose deviations for this group compared to the other patients. Gamma pass rate at the most strict evaluation criteria, 1%, 1 mm of the full dose distribution, was on average 99.1 ± 0.6% and 100 ± 0.0% when restricted to the PTV. None of these differences are relevant in the clinical setting, hence showing that the sCT images of this study are suitable substitutes for CT images in the dose calculation.

In the present study, 14 out of the 20 patients had volumes of bone resection due to surgical procedures prior to radiotherapy. From our evaluation it was evident that the MRI Planner was able to generate sCT images which accurately depicted the anatomy in regions of bone resection. For some patients part of the bone structure in the sCT was a few mm thicker than in the CT, particularly around the resection volume. This anomaly had however no significant effect on the results of the dosimetric evaluation. The resulting MAE for bone and DSC in the VOI surrounding the post-surgical region were within 1 S.D. of those including the whole skull. The results were also comparable to those of the patients with intact skull bone.

Another important evaluation concerns patient positioning at treatment, which in previous work has been shown to be affected by abnormal anatomy [29, 30]. When excluding the CT in an MRI-only workflow, the sCT images become the reference of image guided radiotherapy. The anatomical anomalies due to surgery are often connected or adjacent to the target and therefore commonly used as landmarks when reviewing the registration result. In this study, setup verification differed on sub-mm in translational differences and sub-degrees in rotations between sCT-CBCT and CT-CBCT image registrations, which is well within reported variations of automatic registration methods [31]. No significant difference was observed between patients with and without volumes of bone resection. Hence, patient positioning using MRI Planner generated sCT images as references for CBCT-image registration is feasible in an MRI-only workflow.

Evaluation of sCT for brain with anatomical anomalies have been scarce in literature [4, 5, 32]. A few atlas-based methods have been evaluated but the sCT generation failed to assign the correct HU values in the region where patient anatomy and atlas did not match [7–9]. A more recent study reported a deep-learning-based approach where the sCT images for two patients were visually well-represented in the regions affected by surgery [16]. This study was done on a 1.0 T MR scanner and there was no detailed or quantitative evaluation of the affected regions with respect to geometry or dosimetry.

Previously published studies with deep-learning based methods in the following comparison are based on a T1w sequence, with or without contrast enhancement [11, 13–16, 33], whereas this study uses four-channel Dixon MRI. 4–6 min scanning time has been reported for T1w data acquisition [11, 14], comparable to 4.5 min for our Dixon sequence. Even though this method requires an extra sequence in addition to the clinical MR-protocol, scanning time should not be a major concern given the original protocol being in the time frame of 20 min. The overall results in this study were in line with previously published work, such as our average MAE of 62.2 HU compared to reported MAE from just under 50 HU up to 90 HU [13–16, 33]. The mean target dose differences between the dose distributions calculated on sCT and CT images was 0.1 ± 0.2%, which agrees with previously published values where similar or slightly larger deviations were found [14, 33]. Overlap in bone, DSC, between sCT and CT images in this study was similar or better than previously reported values [14, 15]. Mean patient specific geometric distortion in the MR images used for sCT generation was below 0.1 mm, which is in line with previously reported values [14].

The immobilization mask was not depicted in the sCT images since it did not yield any useful signal in the MR Dixon images. Although present in the CT images of comparison, the absence of a mask in sCT did not constitute a problem because absorbed dose calculation in all images was only performed for the voxels within the body contour and treatment couch structure. Even with the fixation included in the dose calculations, the attenuation of the mask for photon irradiation (6–15 MV) is in the order of 0.5%, according to the manufacturer, which is not a concern for the dose delivered to the patients.

Due to the attenuation of radiation in the treatment couch, the position is necessary for accurate absorbed dose calculations. Since the couch did not provide any signal in the MR Dixon images a ZTE sequences was used to image the couch. Although these images had poor signal to noise ratio, it was sufficient for the purpose and the couch was successfully identified for all patients investigated. In the present work, the top of the treatment couch was identified and the distance between the couch and the lowest part of the patient skull was measured. An alternative method would be to fuse the ZTE image with the sCT in the TPS directly to easily find the correct vertical positon of the inserted couch. By adding the ZTE sequence to the MR protocol, the position of the couch can be retrieved accurately from image data rather than an uncertain manual procedure using a ruler. It also avoids a potential logistics problem transferring the distance information from the person operating the MRI to the person performing the treatment planning.

To focus on the relevant differences between the images during evaluation, the sCT was rigidly registered to the CT in six degrees of freedom, prior to dose calculation. The dosimetric differences that were in fact present, even though small, were therefore a result of the combined errors due to HU offset between sCT and CT images, small geometric distortions in the Dixon images and remaining deviations in positioning between MR and CT examinations (despite registration). A different way to evaluate doses would be to not only recalculate but rather re-optimize the treatment plan on the sCT images, which may result in different dose planning parameters between the two plans. However, this adds another dimension of uncertainty, and was therefore not applied in this study. Re-optimization has been shown to yield similar results to re-calculation [33]. Another approach would be to perform deformable image registrations to further isolate the relevant differences. However, major anatomical variations are improbable in the brain or skull between imaging sessions. Deformable image registration would be more advantageous for other diagnoses such as head and neck cancer, where larger anatomical variations are expected. Hence, our conclusion was that a rigid registration was sufficient for the purpose of this study.

For target delineation in SRS, the recommendation regarding geometric distortions and spatial resolution in MR images is below 1 mm [27]. A 3D based T1w + Gd image series (voxel size 1×1x1mm^3^) for delineation was used in the present study, which fulfils this requirement. However, the Dixon images for sCT generation and dose calculation had 2 mm slice thickness. This is a limitation, but for evaluating a steep gradient dose distribution and CBCT image registrations, as performed in this study, the spatial resolution was sufficient [34, 35]. Thus, the validations performed in this study are applicable also to SRS treatment plans, but to minimize partial volume effects the Dixon protocol should be adjusted to fulfill the requirement of 1 mm slice thickness prior to clinical implementation of MRI-only SRS. 

The long-term goal of this research is to implement the MRI-only workflow for brain tumour radiotherapy using MRI planner for sCT generation at our clinic. This is a somewhat different approach from many of the previous studies, where in-house developed generation methods have been validated showing proof of principle rather than a validation prior to a clinical implementation. Some studies have a limited number of patients compared to this work [29, 36–38] and most of them did not evaluate anatomical anomalies [11, 14, 33]. We have observed that the research field of sCT generation methods for brain in general has progressed with great results. Yet, this is the first clinical evaluation of a CE-marked sCT generation software that enables a wider range of clinics to implement MRI-only radiotherapy planning for brain. We hope that this study will help to promote further use of this technology.

## Conclusions

This is to the best of our knowledge the first study to validate a commercial sCT generation software feasible in the treatment of brain malignancies with and without anatomical anomalies in the skull due to surgery. Comparable results were found between sCT and CT images for both absorbed dose calculations and patient positioning. This study paves the way for a clinical implementation of MRI-only brain radiotherapy.

## Supplementary Information

**Additional file 1**. Complete image dataset.

## Data Availability

The datasets generated during/or analyzed during the current study are not publicly available due to patient privacy concerns and institutional regulations.

## Abbreviations

CBCT: Cone beam computed tomography
CT: Computed tomography
DSC: Dice similarity coefficient
HU: Hounsfield unit
kV: Kilo voltage
MAE: Mean absolute error
ME: Mean error
MR: Magnetic resonance
MRI: Magnetic resonance imaging
OAR: Organs at risk
PTV: Planning target volume
sCT: Synthetic computed tomography
SD: Standard deviation
TFE: Transfer function estimation
TPS: Treatment planning system
uTE: Ultrashort echo time
VMAT: Volumetric modulated arc therapy
VOI: Volume of interest
ZTE: Zero echo time
