# Supplementary material for: Clinical validation of a commercially available deep learning software for synthetic CT generation for brain
Source: Radiat Oncol. 2021 Apr 7;16:66. doi: 10.1186/s13014-021-01794-6 (PMC8025544; doi:10.1186/s13014-021-01794-6)
Supplement: Supplementary file 1 — Additional file 1. Complete image dataset. [file 13014_2021_1794_MOESM1_ESM.docx]

**Supplementary material: Complete image dataset**

*
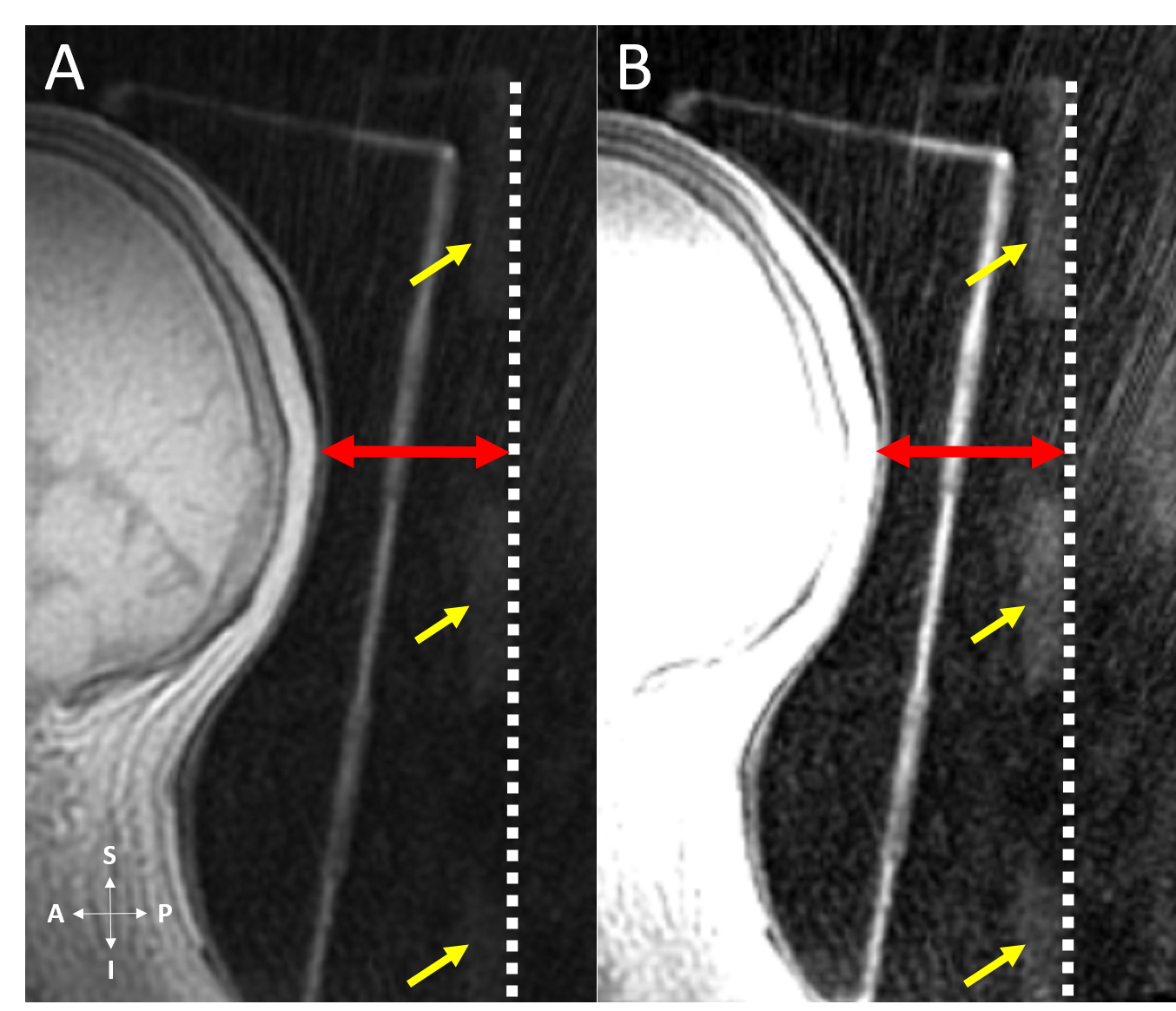
*

**Figure E1.** (A): Representative case of the Zero Echo Time (ZTE) image used for treatment couch identification. Field of view was defined to cover the back of the patient head and to include the table top. (B): The window level has been adjusted to increase the table top visibility (highlighted by the inserted white dotted line). The yellow arrows show the base to which the immobilization mask is fixated, placed on top of the treatment couch. The red arrow indicates the distance between the patient head and the table top.

*
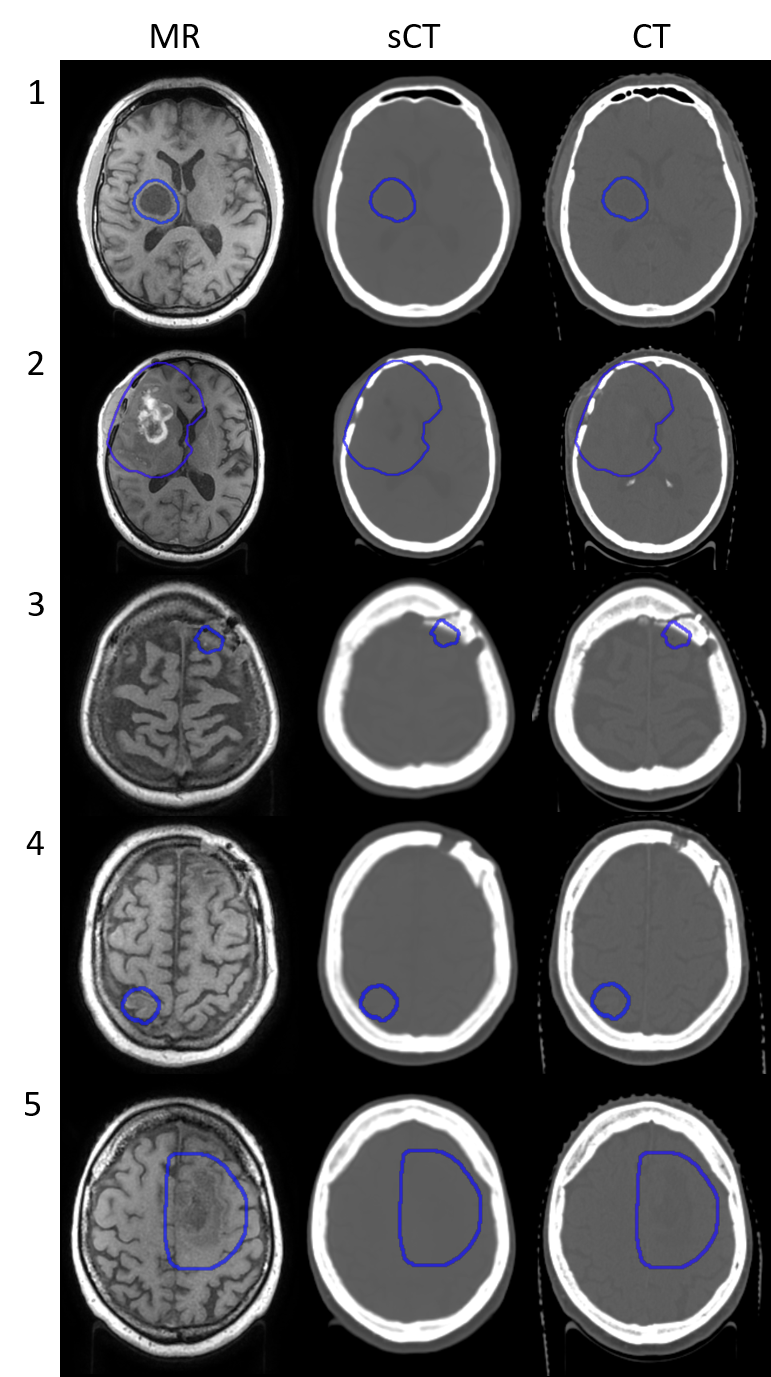
*

**Figure E2.** MR-Dixon (in-phase), synthetic CT and CT images presented in one transversal slice for patient 1-5. The PTV structure for each patient is outlined in blue. Further details about each patient can be found in Table 2 of the main manuscript.

*
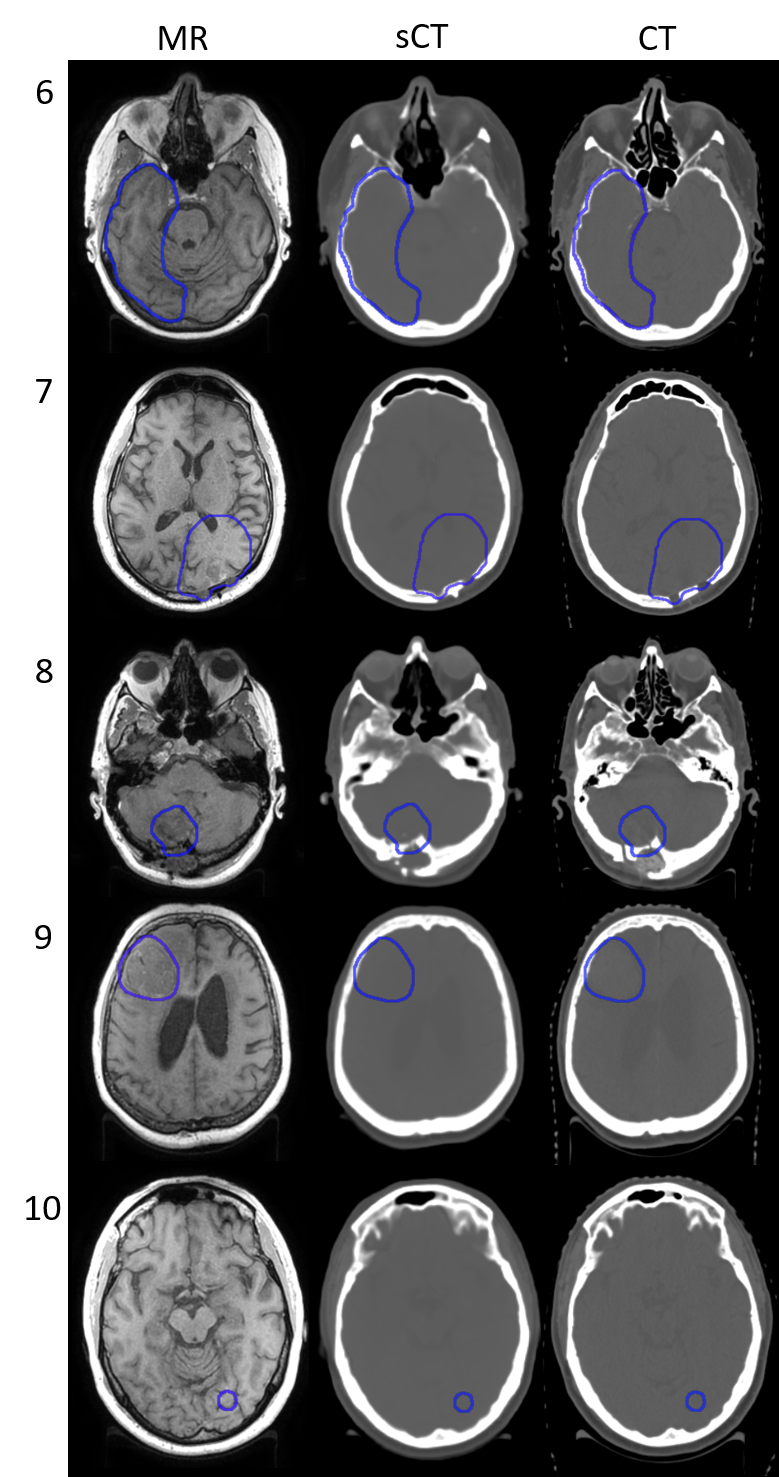
*

**Figure E3.**MR-Dixon (in-phase), synthetic CT and CT images presented in one transversal slice for patient 6-10. The PTV structure for each patient is outlined in blue. Further details about each patient can be found in Table 2 of the main manuscript.

*
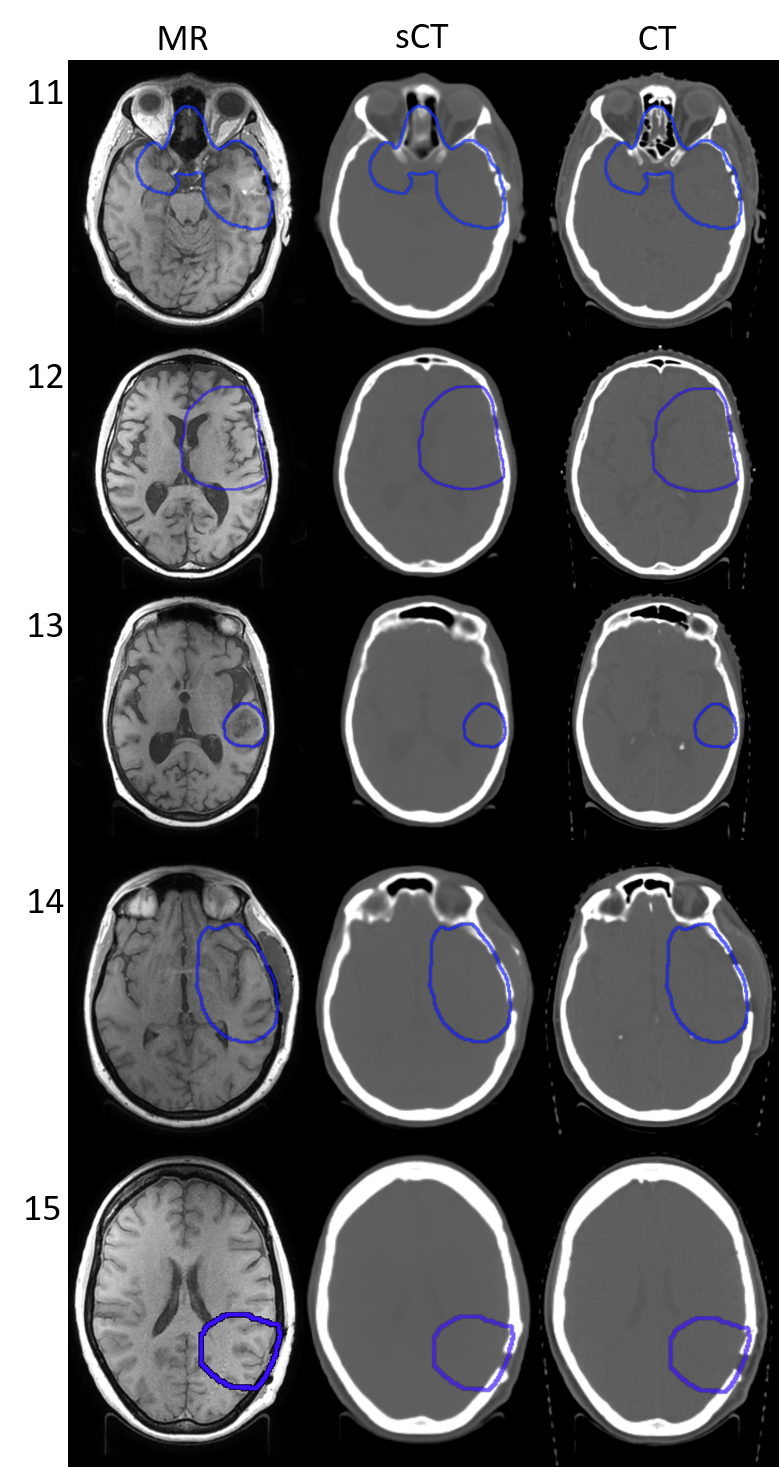
*

**Figure E4.**MR-Dixon (in-phase), synthetic CT and CT images presented in one transversal slice for patient 11-15. The PTV structure for each patient is outlined in blue. Further details about each patient can be found in Table 2 of the main manuscript.

*
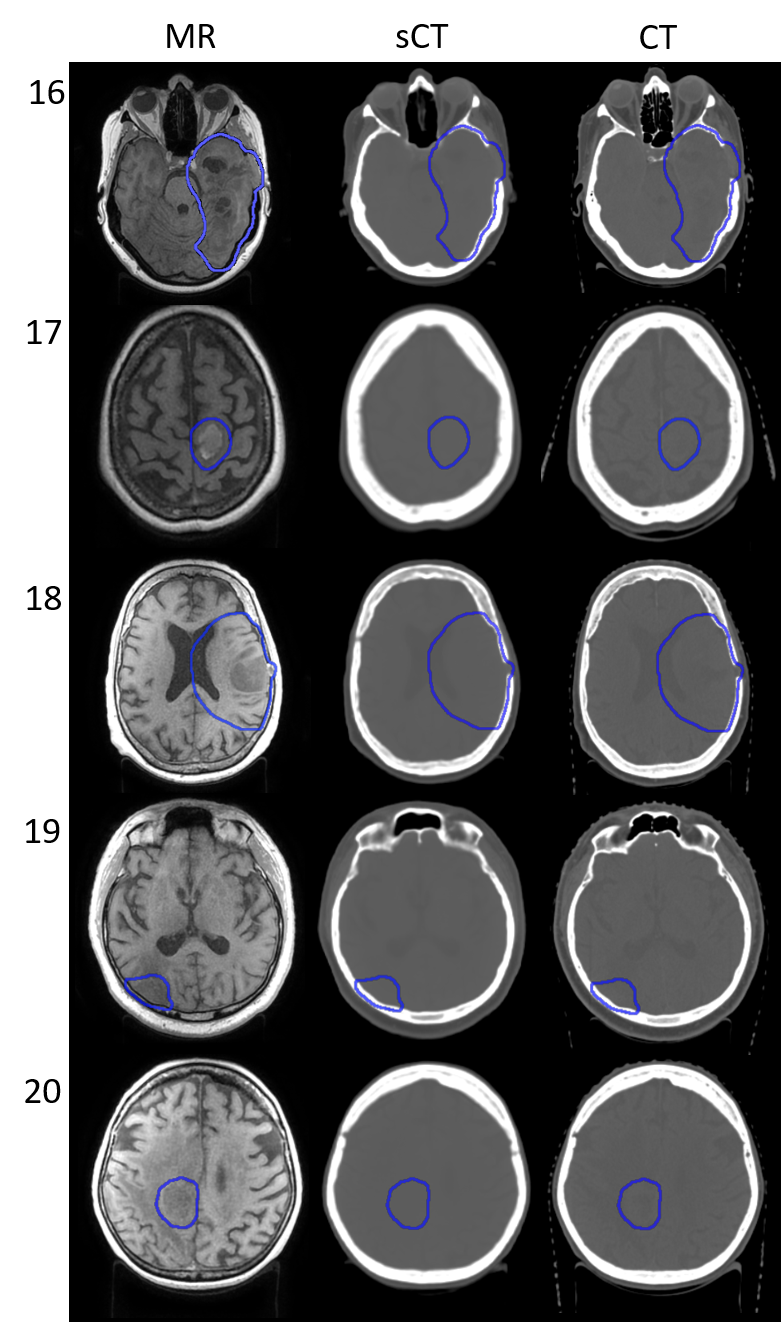
*

**Figure E5.** MR-Dixon (in-phase), synthetic CT and CT images presented in one transversal slice for patient 16-20. The PTV structure for each patient is outlined in blue. Further details about each patient can be found in Table 2 of the main manuscript.
